# Surgical Rescue for Refractory Post-sphincterotomy Hemorrhage: Preserving a Critical, Vanishing Competency for the Next Generation of Surgeons

**DOI:** 10.7759/cureus.113831

**Published:** 2026-08-02

**Authors:** Dheeraj Kethireddy, Suresh Kumar Palaniappa

**Affiliations:** 1 Surgical Gastroenterology, Sri Ramachandra Institute of Higher Education and Research, Chennai, IND

**Keywords:** duodenotomy, ercp complications, post-sphincterotomy hemorrhage, refractory gastrointestinal bleed, surgical education, surgical hemostasis

## Abstract

Post-endoscopic retrograde cholangiopancreatography bleeding is a classic, well-documented clinical hurdle. While contemporary management successfully resolves the vast majority of these episodes via repeat endoscopy or transcatheter arterial embolization, refractory hemorrhage requiring an emergency open operation remains a rare, highly dangerous threat. We report the case of a 64-year-old woman who developed a severe, delayed post-sphincterotomy hemorrhage. The bleeding achieved initial hemostasis with an aggressive mechanical dual-stent tamponade, but the patient subsequently re-bled, completely resisting further endoscopic modalities and empiric endovascular angioembolization using both coils and glue. Driven by rapid clinical decline and unrelenting hemorrhagic shock, the team bypassed further minimally invasive revisions and proceeded immediately to the operating room. Emergent exploratory laparotomy, broad duodenal mobilization (Kocher maneuver), forward duodenotomy, and direct transfixion suture ligation of an actively spurting vessel within the pancreaticoduodenal arcade achieved immediate, definitive hemostasis. The patient recovered smoothly and was discharged without secondary issues. This case strongly reminds the clinical community that while open surgery has largely been supplanted by modern technology, it remains an indispensable, life-saving fallback. Crucially, because the current generation of general and hepatobiliary surgeons seldom encounters this clinical crisis during training, preserving technical familiarity with open ampullary vascular control is an essential educational priority to prevent catastrophic patient outcomes when minimally invasive choices fail.

## Introduction

Endoscopic retrograde cholangiopancreatography (ERCP) is a foundational tool in the modern management of complex pancreatobiliary diseases. Over the past few decades, its role has fundamentally shifted from a diagnostic test to a highly technical therapeutic intervention. This shift brings an inherent risk profile, with overall adverse events occurring in 5% to 10% of cases [1,2]. Among these, post-sphincterotomy hemorrhage is one of the most clinically alarming, complicating roughly 0.5% to 2.0% of procedures as cited [3-5]. While minor mucosal oozing immediately following a sphincter cut frequently resolves on its own or with quick thermal intervention, severe, delayed bleeding can trigger abrupt hemodynamic collapse, catching clinicians off guard days after an apparently successful procedure.

First-line management rests on urgent repeat endoscopy, deploying combined interventions such as epinephrine injection, thermal coagulation, hemoclips, over-the-scope clips, topical hemostatic agents, or advanced mechanical maneuvers like fully covered self-expandable metal stents (cSEMS) or multi-plastic stent placement to physically compress the bleeding site [6]. When endotherapy fails to stabilize the patient, interventional radiology provides an excellent secondary defense, utilizing transcatheter arterial embolization to map and occlude the broken vessel.

Because these advanced endoscopic and radiologic tools boast incredibly high success rates, the actual requirement for an emergency surgical rescue has fallen to a fraction of a percent [7,8]. Consequently, open duodenotomy for a bleeding ampulla has virtually disappeared from modern surgical residency logbooks. A new generation of general and hepatobiliary trainees can easily finish their training without ever seeing or performing these delicate, high-stakes open procedures. Yet, when a patient bleeds through both optimal endoscopy and multi-arcade embolization, time runs out, and an immediate open rescue becomes a critical, life-saving intervention.

The clinical significance and precise novelty of this case reside in the documented survival of a patient after the concurrent failure of both advanced double-plastic biliary stenting, mechanical tamponade, and endovascular dual-arcade coil and cyanoacrylate glue embolization, necessitating an immediate transition to open surgical rescue. This case demonstrates that sometimes there is a limit to modern endovascular and endoscopic hemostatic algorithms, proving that the rare need for manual, open transfixion ligation remains an indispensable life-saving fallback when the complex collateralization of the pancreaticoduodenal vascular network permits hemodynamic escape.

## Case presentation

A 64-year-old female patient arrived at our emergency department in profound hemorrhagic shock, presenting with sudden, copious hematemesis and melena. Her medical history was notable for a recent therapeutic ERCP and biliary sphincterotomy performed at an outside facility for symptomatic choledocholithiasis. On arrival, she was pale, diaphoretic, and severely hypotensive. On arrival, the patient's hemoglobin was 4.5 g/dL. The clinical team immediately started aggressive fluid resuscitation and typed blood transfusions; two units of packed red blood cells (PRBCs) were given while concurrently planning for upper gastrointestinal endoscopy to identify and address the source of the bleeding.

Endoscopic intervention

**Figure 1:**
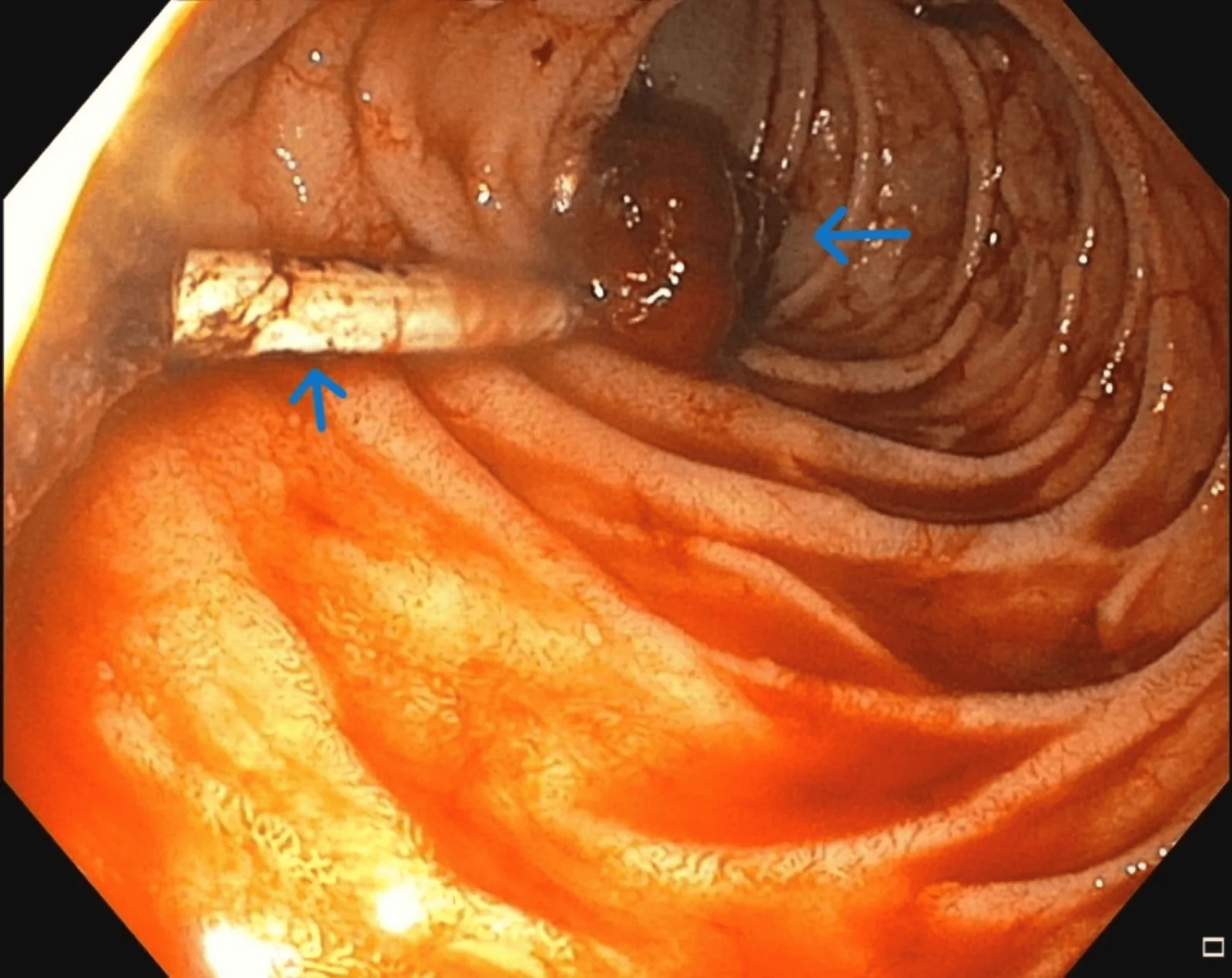
Endoscopy image showing stent and blood clots Initial endoscopic view of the major duodenal papilla showing a large, adherent sentinel blood clot (right arrow) over the sphincterotomy site with active pooling of blood in the duodenum. A pre-existing plastic stent is visible in situ (upward arrow).

**Figure 2:**
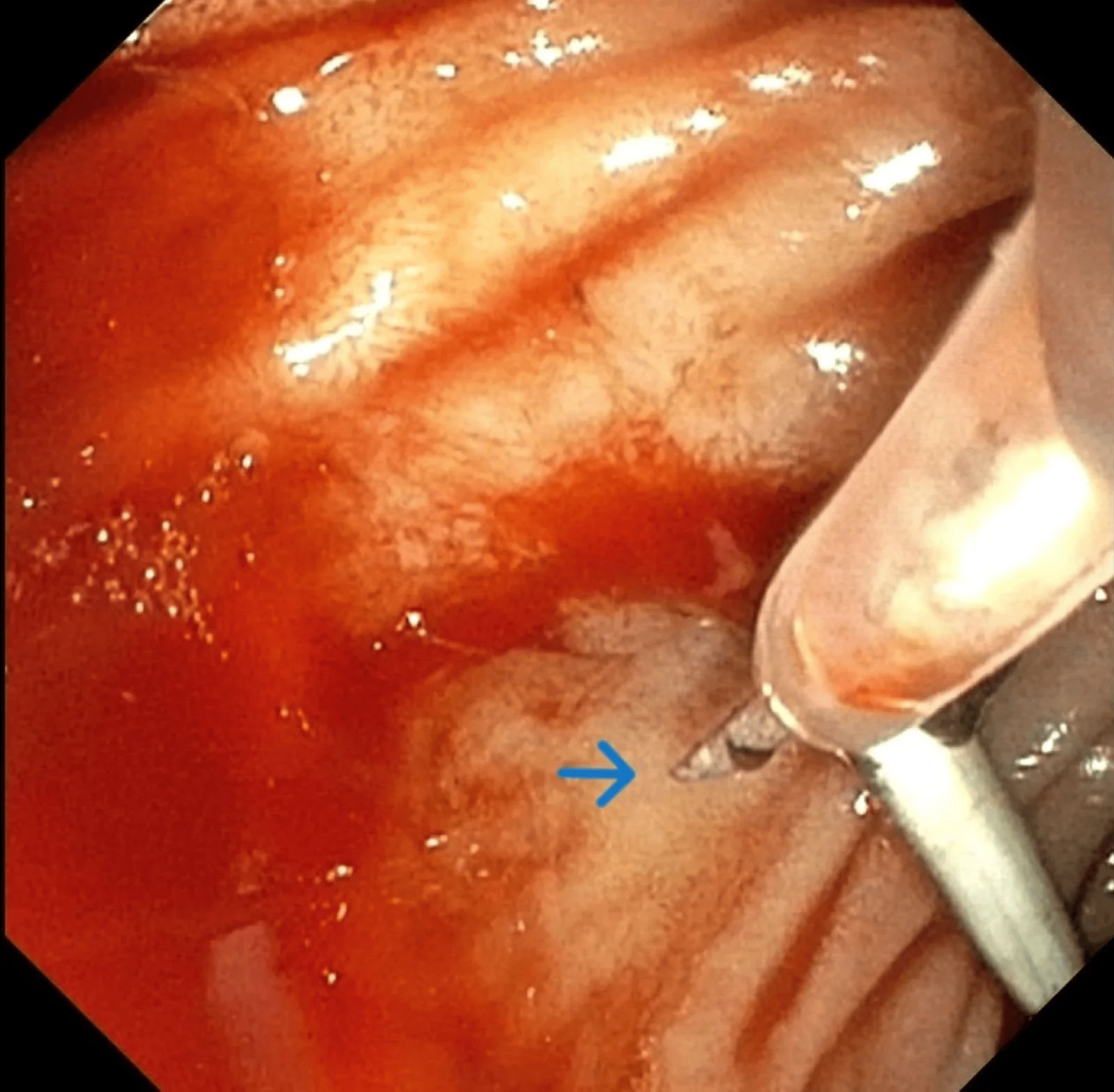
Endoscopic image showing the thermal coagulation probe Application of thermal hemostasis. A coagulation catheter is positioned to deliver targeted electrocoagulation to the bleeding vessel at the sphincterotomy margin.

**Figure 3:**
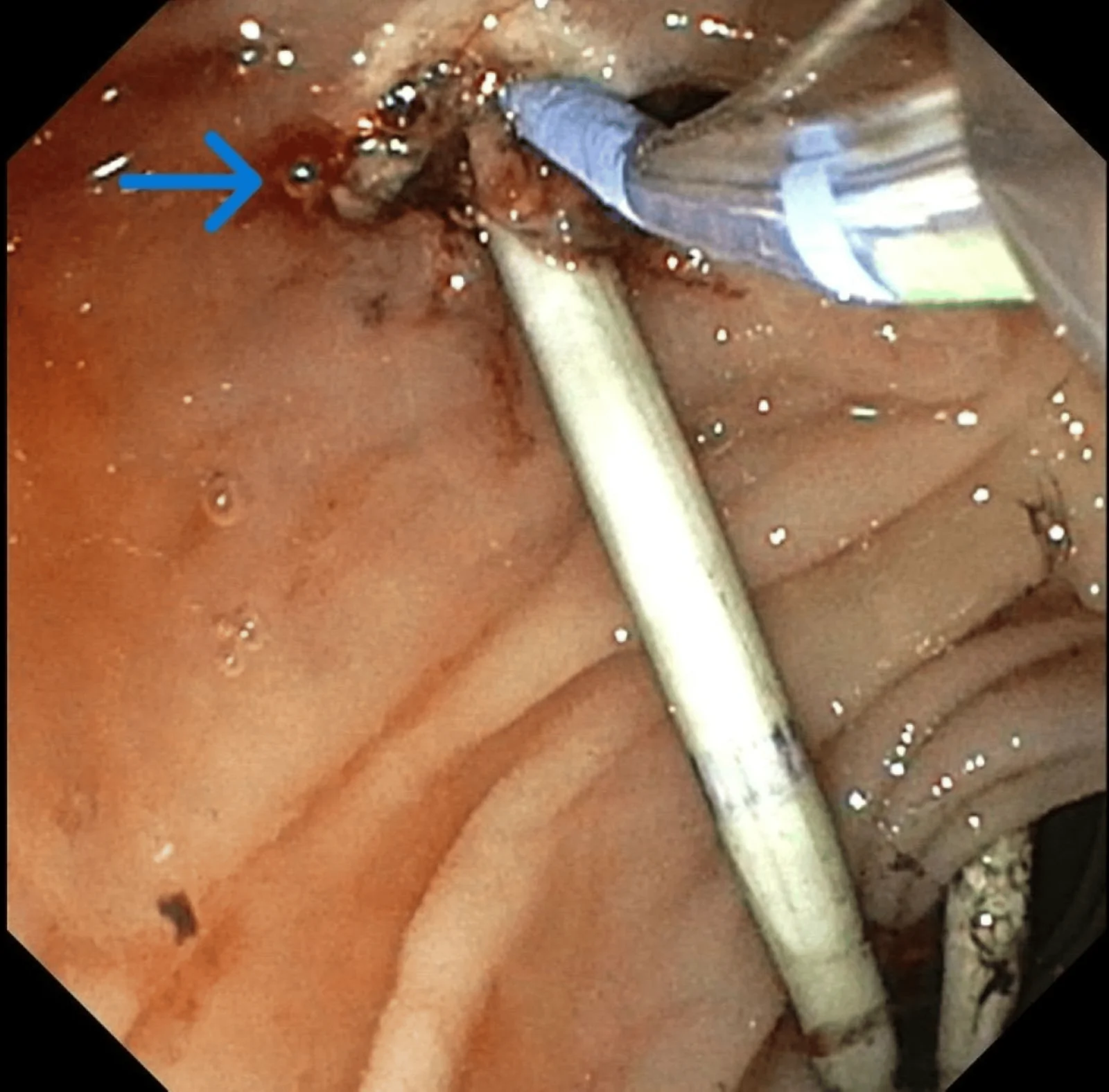
Final endoscopic showing successful double plastic stenting The placement of two side-by-side stents provides mechanical tamponade against the mucosal incision edges. Mild tissue blanching and charring from the thermal coagulation are visible at the superior margin (blue arrow).

Upon introducing the duodenoscopy into the second part of the duodenum, the team encountered a massive, adherent clot with active, brisk arterial blood oozing from the upper apex of the prior sphincterotomy site (Figure 1). The previous stent was removed to access the bleed. The clinical team systematically executed a series of aggressive hemostatic steps: first, 2 mL of 1:10,000 epinephrine was locally injected into the apex of the cut to promote rapid vasoconstriction and local tissue edema. Because this only slowed the flow slightly, a direct contact heater probe was applied, achieving only partial, unstable control. The common bile duct (CBD) was then successfully cannulated, allowing for targeted electrocoagulation directly to the bleeding margins via a thermal probe (Figure 2). To optimize mechanical control, the team implemented an aggressive double-stenting mechanical tamponade strategy [3]. A 10 Fr x 10 cm straight CBD stent was deployed across the ampulla, immediately followed by a second, adjacent plastic biliary stent (Figure 3). This dual-stent construct created tight radial compression against the margins of the sphincterotomy cut, successfully halting the active ooze and providing initial endoscopic hemostasis.

Clinical refractory course and angioembolization failure

**Figure 4:**
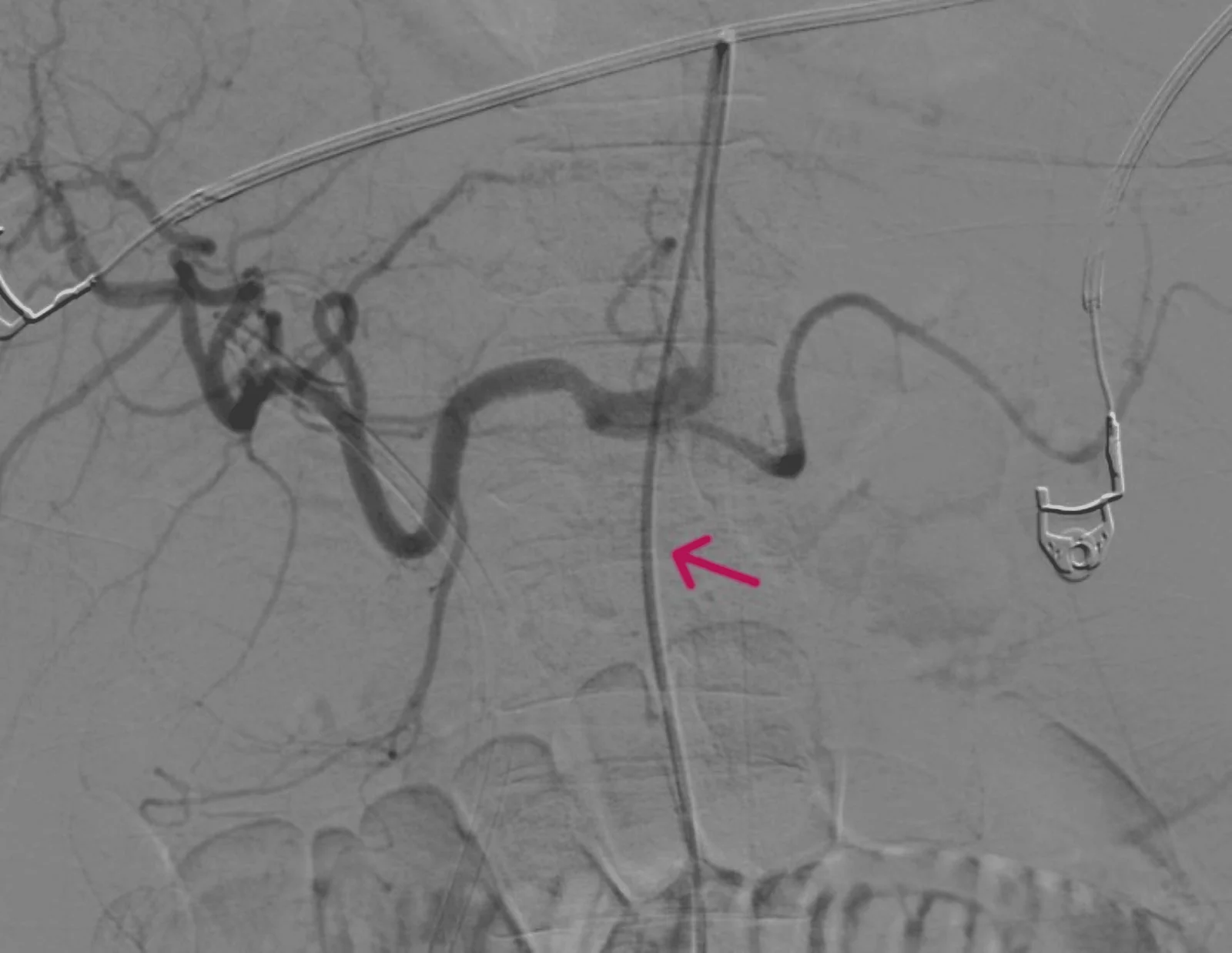
Digital subtraction angiography (DSA) of the celiac trunk demonstrating the visceral arterial anatomy, including the gastroduodenal artery (GDA) (red arrow) and pancreaticoduodenal arcade prior to intervention Crucially, there is no macroscopic contrast extravasation or pseudoaneurysm identified, a key negative finding that necessitated the subsequent empiric embolization strategy.

**Figure 5:**
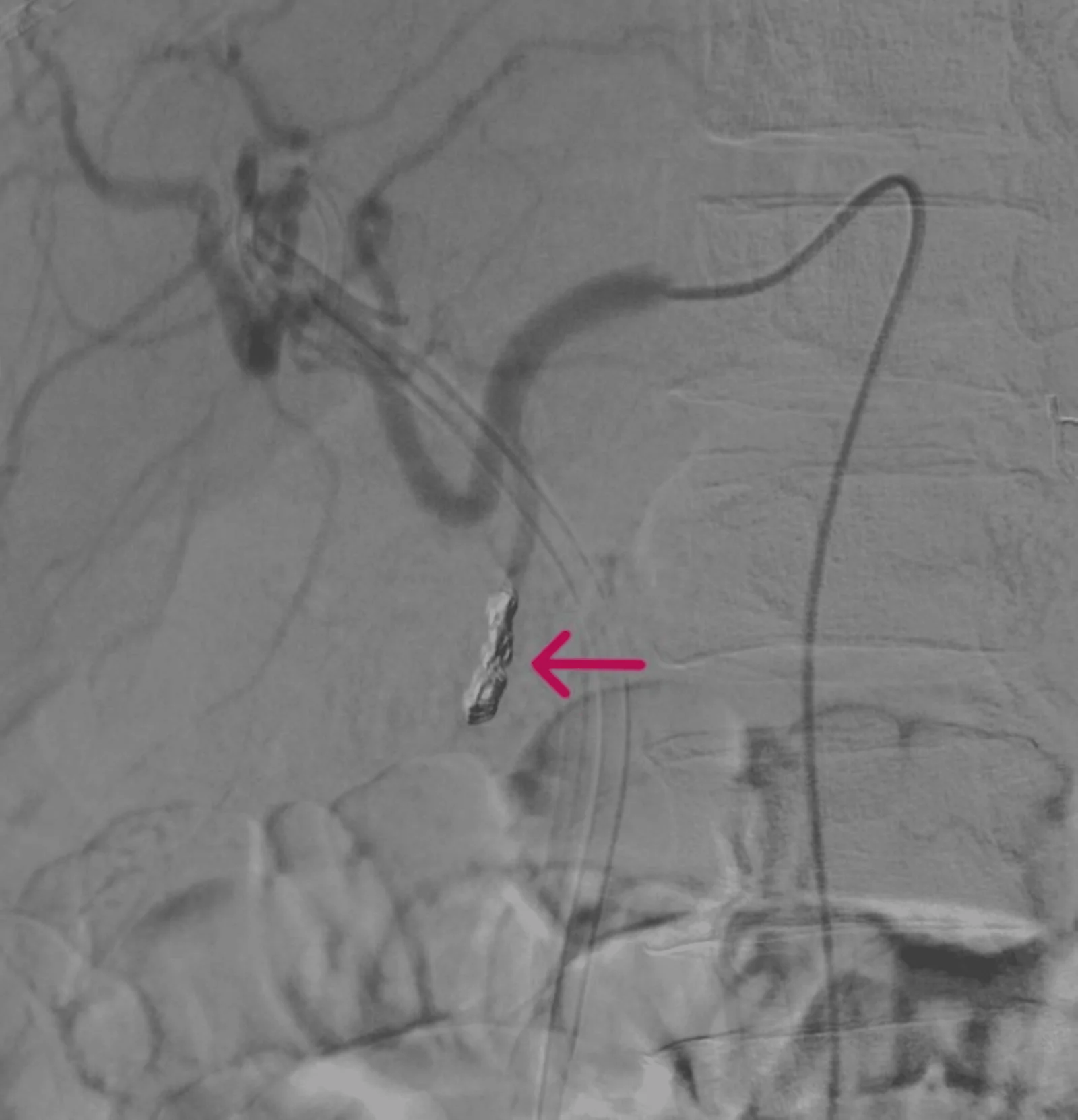
Post-embolization completion digital subtraction angiogram image with coil Post-embolization digital subtraction angiogram demonstrating empiric coil embolization of the gastroduodenal artery. A dense coil pack is visible within the proximal GDA, successfully occluding the forward arterial flow, with preserved contrast opacification of proper hepatic artery branches. GDA: gastroduodenal artery

**Figure 6:**
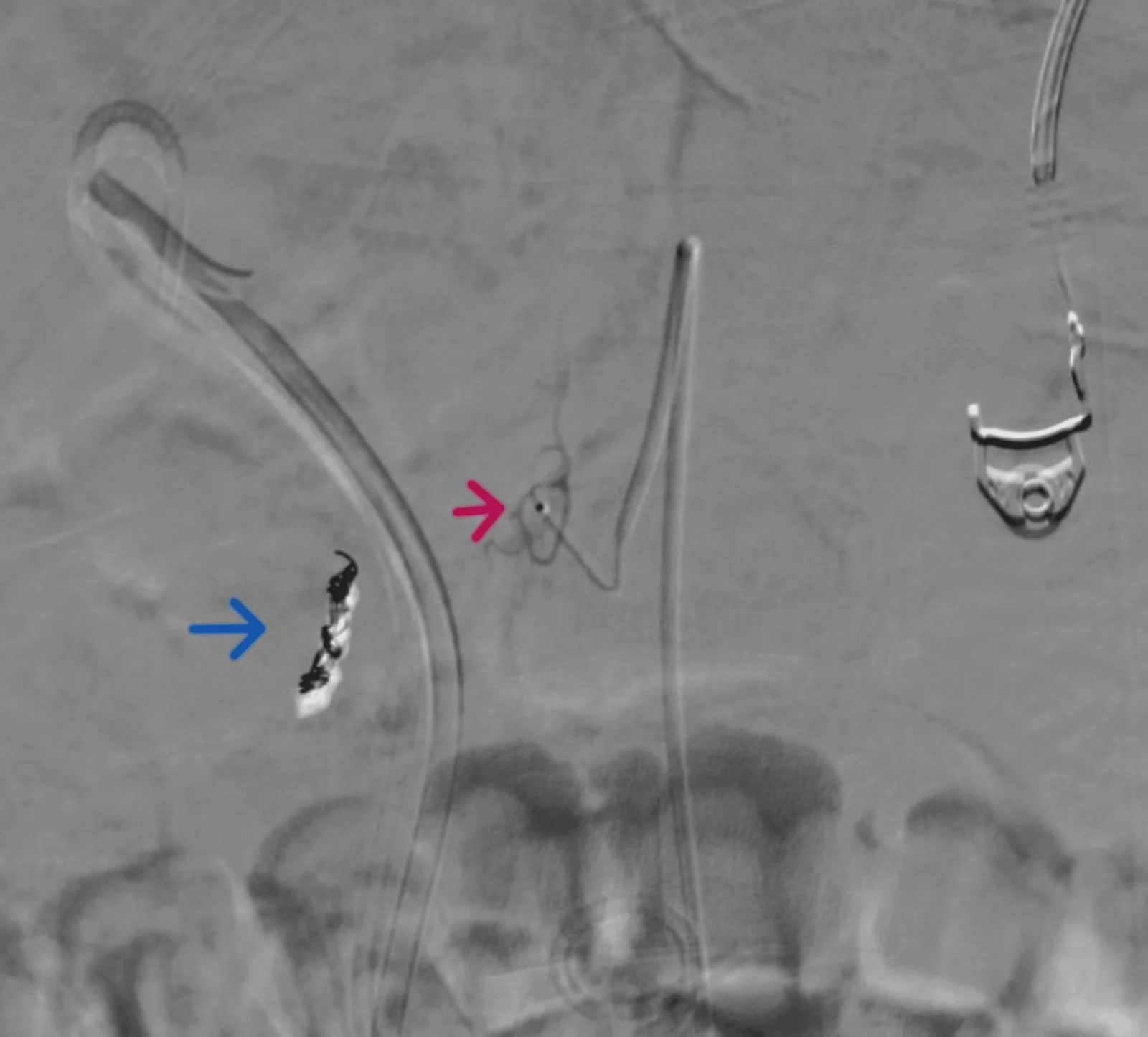
Post-intervention fluoroscopic image documenting the dual-arcade embolization. The metal coil pack visible within the gastroduodenal artery (blue arrow), alongside N-butyl cyanoacrylate (nBCA) glue mixture (red arrow) successfully deployed within the inferior pancreaticoduodenal artery

The patient was transferred to the intensive care unit (ICU) for vigilant monitoring. Unfortunately, the bleeding never fully ceased clinically. Within hours, the patient experienced recurring bouts of hematemesis and persistent melena, requiring continuous PRBC transfusions to keep pace with an ongoing drop in hemoglobin and volatile vitals. Given the recurrence of bleeding, the team planned for metal stent placement but deferred in our case due to rapid hemodynamic deterioration and recurrent and massive hematemesis will make upper endoscopy too dangerous regarding aspiration and blind navigation for stent placement; hence, the multidisciplinary team agreed and requested an emergent Interventional Radiology consult for angioembolization [3,6]. Under monitored anesthesia care, retrograde right femoral arterial access was secured. Selective angiography of the celiac trunk, common hepatic artery, and superior mesenteric artery (SMA) was meticulously performed. Figure 4 showed normal vascular anatomy without a clear, macroscopic contrast leak or pseudoaneurysm from the gastroduodenal artery (GDA) or its branches, a common hurdle in intermittent gastrointestinal bleeding. Recognizing the life-threatening nature of the situation, the interventional team elected to perform an empiric dual-arcade embolization. The GDA was selectively cannulated and permanently occluded using 4 mm x 14 mm Nester coils (Figure 5). Following this, the inferior pancreaticoduodenal artery (IPDA) was super-selectively cannulated via the SMA and embolized with a 33% N-butyl cyanoacrylate (nBCA) glue mixture, angiographically isolating the primary blood supplies of the pancreaticoduodenal arcade (Figure 6; Table 1).

**Table 1:** Serial laboratory and coagulation values throughout clinical course INR: International normalized ratio

Clinical Timepoint	Hemoglobin (g/dl)	Platelet Count (x 10^9/L)	INR
Emergency department arrival (day 0)	4.5	170	1.0
Pre-angioembolization	6.5	150	0.9
Post-operative (ICU day 1)	8.7	180	1.1
Prior to discharge	8.9	185	1.1

Emergency surgical rescue

**Figure 7:**
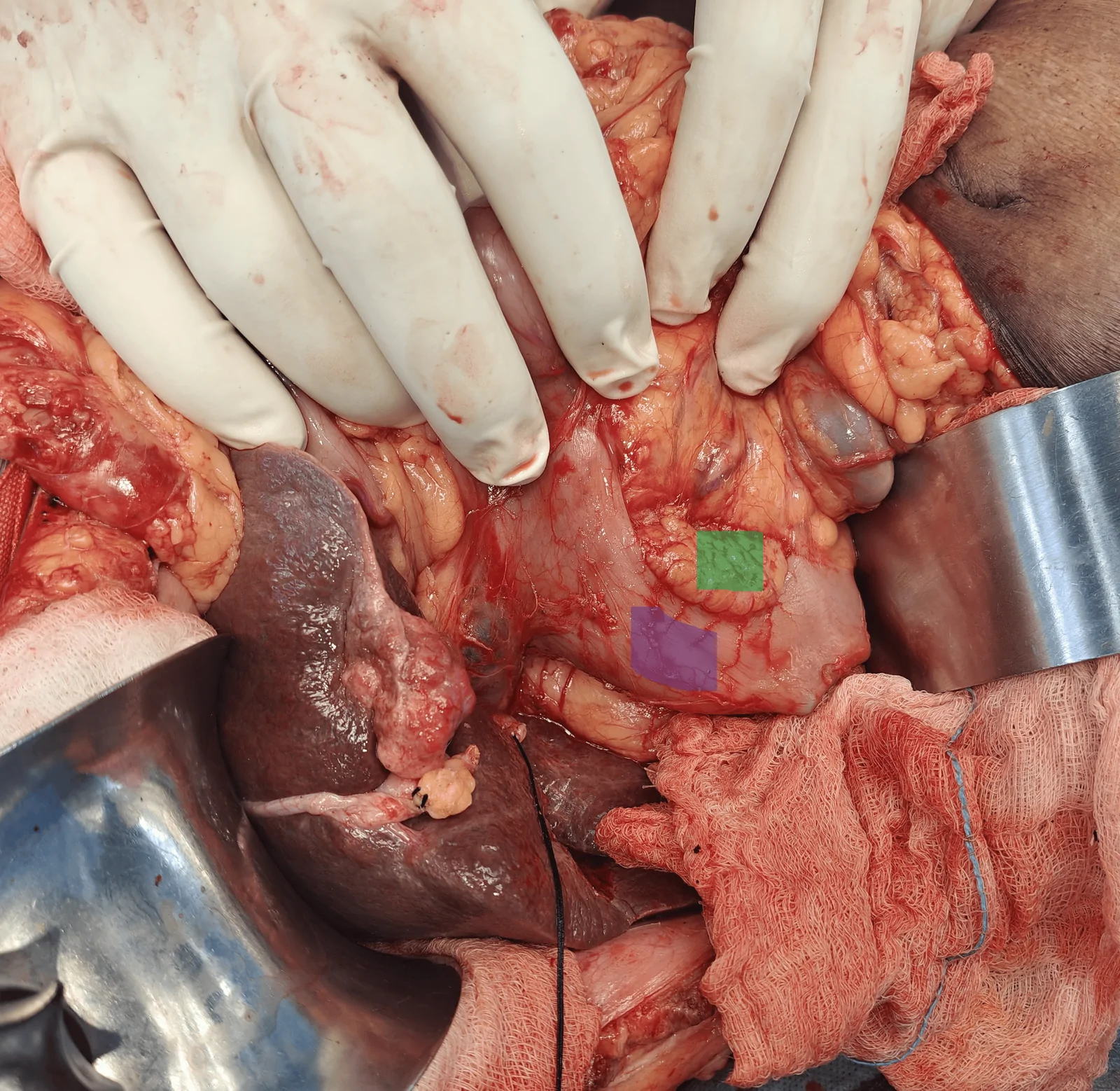
Kocher maneuver: intraoperative visualization of the mobilized second part of the duodenum before duodenotomy Pancreas marked with green shade and duodenum with purple shade

**Figure 8:**
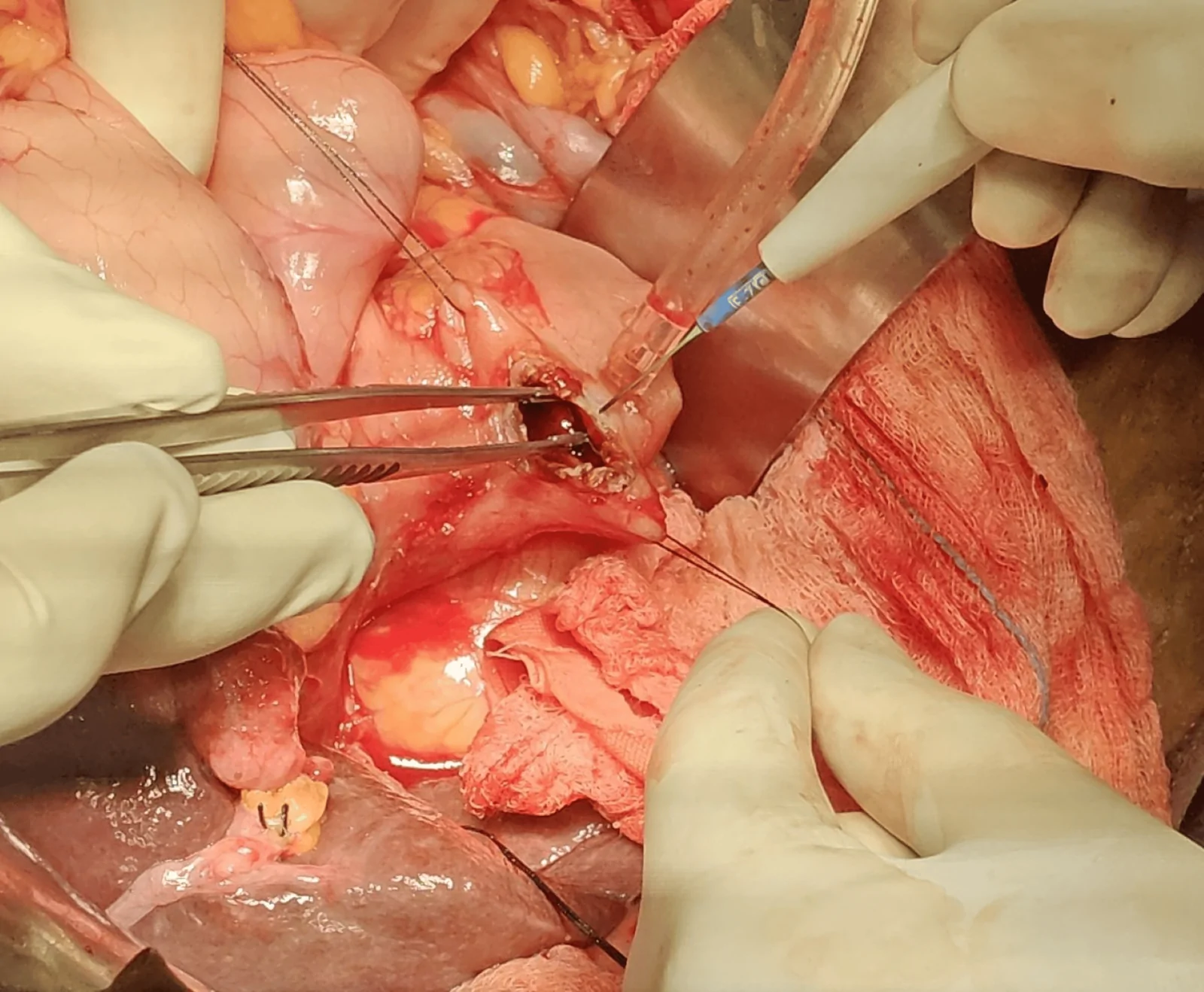
Duodenotomy: initial exposure of duodenal lumen

**Figure 9:**
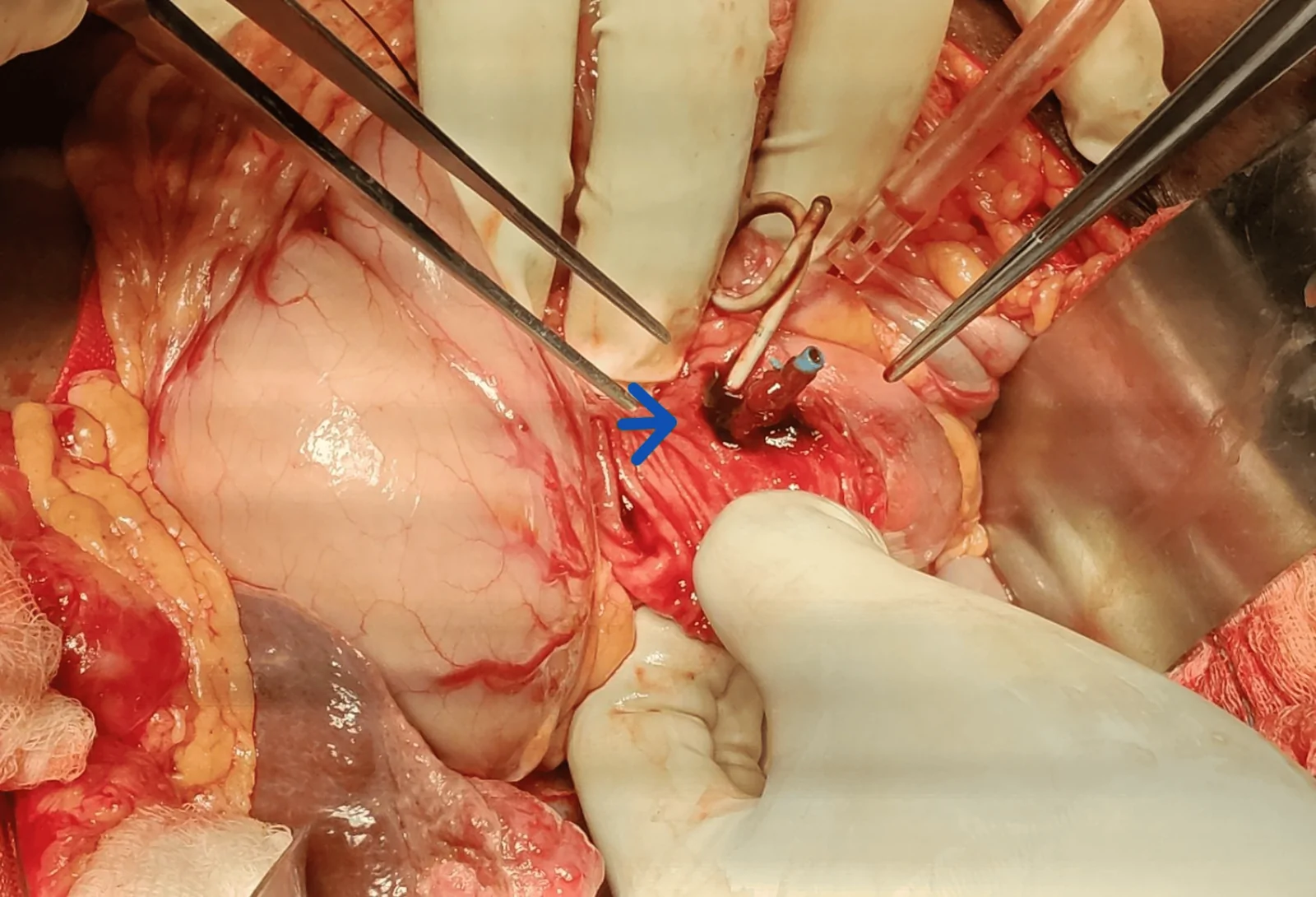
Visualization of the bleeding source: view showing active arterial hemorrhage (marked with an arrow) adjacent to the previously placed double biliary stents

**Figure 10:**
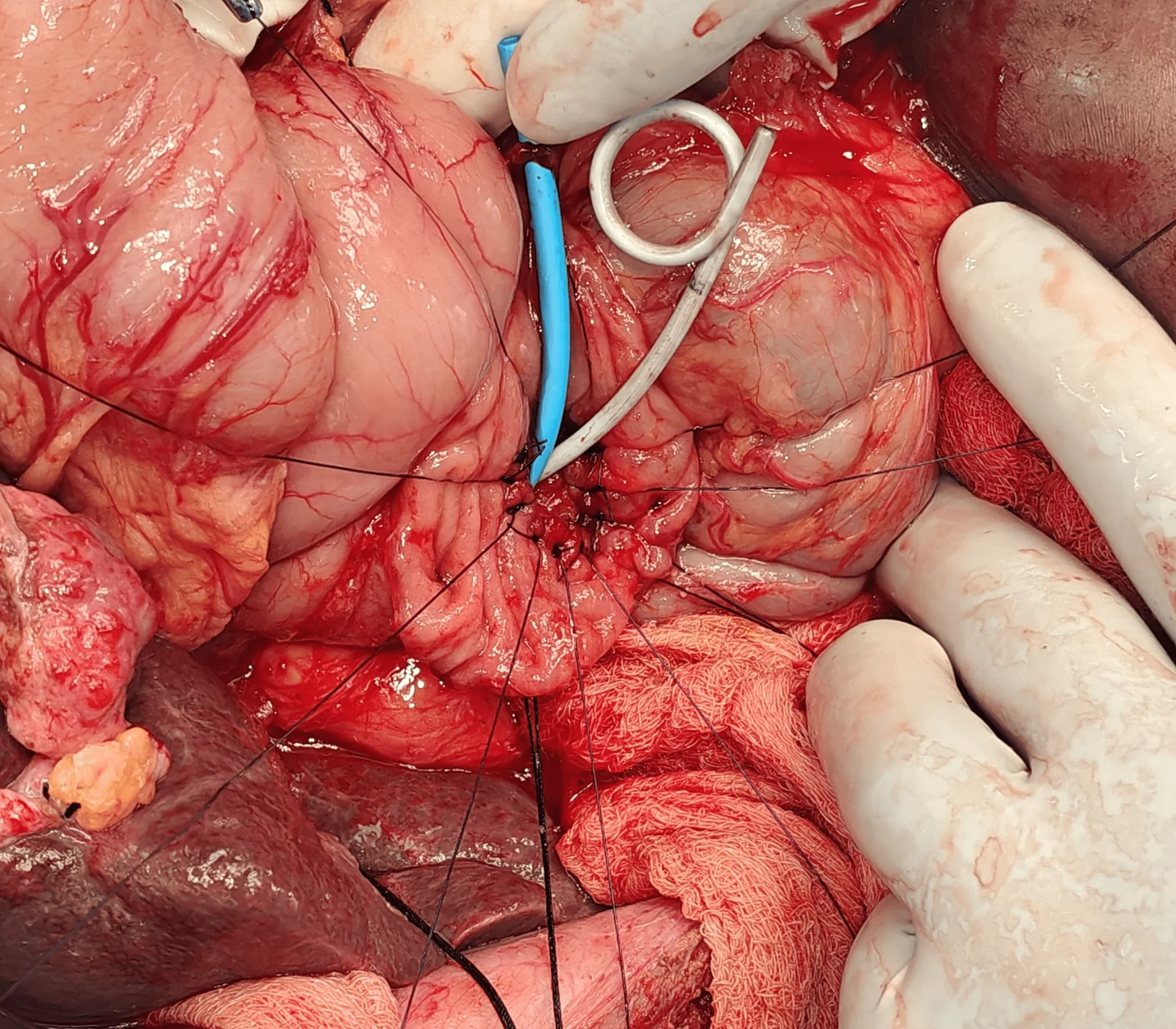
Hemostatic ligation Transfixion suture ligation being placed directly into the apex of the sphincterotomy site and all around to achieve vascular control.

**Figure 11:**
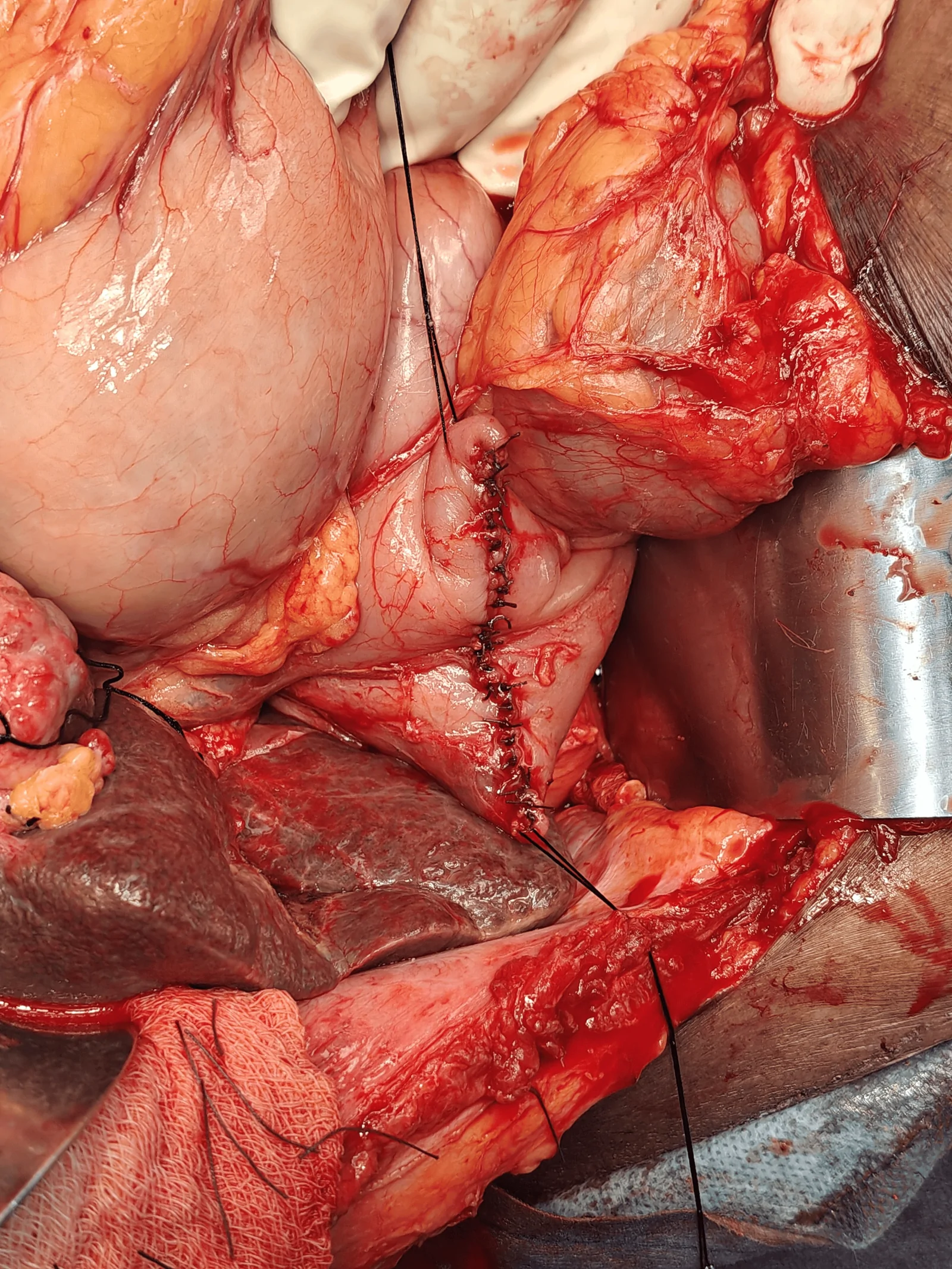
Duodenotomy closure Complete, tension-free closure of the duodenotomy following confirmation of definitive hemostasis.

Despite maximal endoscopic dual-stenting and empiric dual-artery coil and glue embolization, the patient's condition worsened in the ICU. She continued to bleed actively, requiring continuous, high-dose vasopressor support to maintain a MAP above 65 mmHg. With every modern, minimally invasive tool completely exhausted, the patient was taken emergently to the operating room for a life-saving surgical rescue. Through a standard exploratory laparotomy, the surgical team performed a broad Kocher maneuver to fully mobilize the second and third parts of the duodenum (Figure 7). A horizontal forward duodenotomy was executed directly over the mobilized segment (Figure 8). Upon entering the lumen, a substantial volume of fresh blood and organized clots was evacuated. Once the field was optimized, the previously deployed dual plastic biliary stents were visualized. Directly at the superior apex of the prior sphincterotomy cut, the team identified a bleed from the sphincter upper border (Figure 9). This bleeding vessel had clearly drawn from robust collateral pathways within the pancreaticoduodenal arcade, completely bypassing the prior endovascular occlusion. The surgeon placed definitive transfixion hemostatic stitches directly across the apex vessel, achieving immediate, absolute mechanical hemostasis (Figure 10). The patient's blood pressure responded immediately, and she was rapidly weaned off vasopressors on the operating table. The duodenotomy was closed carefully in two layers (Figure 11), and the patient returned to the ICU in stable condition. Her postoperative recovery was entirely smooth and uneventful, and she was discharged home in excellent health (Table 2).

**Table 2:** Timeline of interventions, resuscitation, and outcomes ERCP: endoscopic retrograde cholangiopancreatography; ED: emergency department; GDA: gastroduodenal artery; IPDA: inferior pancreaticoduodenal artery; nBCA: N-butyl cyanoacrylate.

Timeline (Days)	Clinical Event	Key Findings, Interventions, and Support
Day 5	Index procedure	Therapeutic ERCP and biliary sphincterotomy performed at outside facility
Day 0	ED presentation	Arrived in severe shock (Hb 4.5 g/dl) resuscitated with two units of PRBCs, started on vasopressor support with noradrenaline
Day 0	Endoscopic rescue	Active arterial ooze identified. Interventions: local epinephrine, thermal coagulation and dual 10 fr plastic biliary stents placed.
Day 0 (+ 10 hrs)	Rebleeding and angiography	Recurrent hematemesis, unstable general condition. Emergent IR coil embolization of GDA and nBCA glue embolization of the IPDA
Day 1	Surgical rescue	Ongoing shock on high-dose vasopressors. Emergent open duodenotomy and transfixion suture ligation
Day 2	ICU step down	Patient stabilized and transferred to ward. Total PRBCs transfused: six units.
Day 9	Hospital discharge	Discharged home in stable condition. Total length of stay: nine days.

## Discussion

Severe, delayed post-sphincterotomy hemorrhage is driven by specific tissue mechanics [3,4]. It usually occurs when a deep, electrosurgical burn extends through the duodenal mucosa and progressively erodes into a branch of the superior or inferior pancreaticoduodenal artery [3,4]. As the sloughing of the initial surgical eschar occurs days later, a high-pressure arterial system is suddenly exposed to the duodenal lumen. The vascular network supplying the head of the pancreas and the duodenum forms a dense, highly collateralized arcade. This anatomic reality explains why single-vessel interventions occasionally fail: even when main trunks like the gastroduodenal artery are angiographically occluded, retro-perfusion from collateral channels can maintain an active, high-pressure spurt at the ampullary cut. This case illustrates the exhaustive limits of modern, minimally invasive algorithms [5]. Endoscopic double-stenting is an excellent mechanical rescue option. While guidelines recommend fully cSEMS for refractory bleeding [3,6], a dual-plastic stent construct was utilized here as an accessible alternative; by placing two rigid plastic stents side by side, the endoscopist builds enough radial force to physically sandwich the bleeding vessel against the rigid duodenal wall. Similarly, empiric angioembolization is a highly effective secondary defense when a clear contrast leak cannot be seen, aiming to strip the arcade of its head pressure [3,8]. However, as this case demonstrates, collateral flow can sometimes bypass even the most aggressive combination of endovascular coils and glue, leaving open surgery as the final line of defense.

The defining value of this case lies in its lessons for modern surgical education. Because advanced endoscopy and interventional radiology are highly successful, the classic open duodenotomy for a bleeding papilla has become exceedingly rare [7,8]. While this decline is great for patients, it creates a dangerous competency gap in training. An entire generation of young surgeons is entering practice without ever experiencing the specific challenges of this operation: the tactical need for complete duodenal mobilization, the precise tactile feel of a forward duodenotomy, and the technical skill needed to throw accurate transfixion stitches inside an actively hemorrhaging, blood-filled ampullary bed. While this single case report cannot represent all instances of refractory bleeding, documenting these rare operations remains vital to ensure that when all modern, technological options fail, the modern surgeon knows exactly how to step in and save a life with basic, manual open surgical skills.

## Conclusions

Refractory post-ERCP hemorrhage is a life-threatening emergency that can occasionally bypass standard minimally invasive protocols. As this case demonstrates, an aggressive dual-stent endoscopic tamponade and empiric multi-vessel endovascular occlusion may still fail due to the highly collateralized nature of the pancreaticoduodenal vascular arcade. Early recognition of this failure and a decisive shift to an emergency open duodenotomy remain vital to preventing catastrophic outcomes. Because the current generation of surgeons rarely encounters this scenario, this case underscores the importance of maintaining technical familiarity with open ampullary mobilization and direct vascular transfixion as a valuable fallback option in surgical training curricula.
